# Mendelian randomization analyses of genetically predicted circulating levels of cytokines with risk of breast cancer

**DOI:** 10.1038/s41698-020-00131-6

**Published:** 2020-09-01

**Authors:** Shen Li, Yan Xu, Yao Zhang, Lili Nie, Zhihua Ma, Ling Ma, Xiaoyu Fang, Xiangyu Ma

**Affiliations:** 1 The second clinical college, Chongqing Medical University, Chongqing, China; 2 Department of Breast and Thyroid Surgery, Daping Hospital, Third Military Medical University, Chongqing, China; 3 Department of Epidemiology, College of Preventive Medicine, Third Military Medical University, Chongqing, China; 4 Student Brigade, College of Basic Medicine, Third Military Medical University, Chongqing, China; 5 Department of Anaesthesia, The first affiliated hospital of Third Military medical University, Chongqing, China; 6 Banan People’s hospital of Chongqing, Chongqing, China; 7 College of public health, Southwest medical University, Luzhou, China

**Keywords:** Breast cancer, Risk factors

## Abstract

To determine whether genetically predicted circulating levels of cytokines are associated with risk of overall breast cancer (BC), estrogen receptor (ER)-positive and ER-negative BC, we conducted two-sample MR analyses using data from the most comprehensive genome-wide association studies (GWAS) on cytokines in 8293 Finnish participants and the largest BC GWAS from the Breast Cancer Association Consortium (BCAC) with totally 122,977 BC cases and 105,974 healthy controls. We systematically screened 41 cytokines (of which 24 cytokines have available instruments) and identified that genetically predicted circulating levels (1-SD increase) of MCP1 (OR: 1.08; 95% CIs: 1.03–1.12; *P* value: 3.55 × 10^−4^), MIP1b (OR: 1.02; 95% CIs: 1.01–1.04; *P* value: 2.70 × 10^−3^) and IL13 (OR: 1.06; 95% CIs: 1.03–1.10; *P* value: 3.33 × 10^−4^) were significantly associated with increased risk of overall BC, as well as ER-positive BC. In addition, higher levels of MIP1b and IL13 were also significantly associated with increased risk of ER-negative BC. These findings suggest the crucial role of cytokines in BC carcinogenesis and potential of targeting specific inflammatory cytokines for BC prevention.

## Introduction

Breast cancer (BC) remains the main cause of cancer death and the second most common female cancer in western countries, although the death rate of BC has dropped by 40% from 1989 to 2016^1,2^. Inflammatory mechanisms have been implicated in genesis, development and metastasis of BC^3,4^, and finally the prognosis and recurrence of BC^5–7^. Cytokines have indispensable functions in pathology of inflammation^8,9^ and could serve as the potential targets of anti-inflammatory intervention of BC.

The role of cytokines in BC carcinogenesis has been widely explored by observational studies and yielded equivocal results, with suggestive associations between risk of BC and abnormal levels of cytokines, including tumor necrosis factor-alpha (TNF-α), interleukin-6 (IL6), C-reactive protein (CRP), transforming growth factor β (TGF-β)^10–13^, etc. However, these relationships between inflammatory cytokines and BC risk identified in observational studies could be susceptible to confounding factors, limited follow-up time, small sample size and the reverse causation, which might mislead the conclusions^14^. For example, contrary to previous studies, plasma CRP level was not found to be significantly associated with BC risk in the Women’s Health Study with 27,919 participants^15^. In addition, both serum levels of IL6 and TNF-α were associated with either increased or decreased risk of BC in different samples^16–19^. Hence, the potential causality of individual cytokines in determining BC risk remains elusive.

One more robust method being widely for causal inference is Mendelian randomization (MR), which could overcome the limitations of observational research^20,21^. MR uses genetic variants as instrumental variables, avoiding being misled by confounding factors, limited follow-up time, small sample size and the reverse causation of different traits^14,20^. Genome-wide association studies (GWAS), which have identified thousands of variants associated with complex traits, bring the wide usage of MR to a new climax^22,23^. Recently, a comprehensive MR analysis regarding genetically determined levels of circulating cytokines and risk of stroke gave us a good demonstration^24^. It was implemented leveraging the most comprehensive GWAS which evaluated 41 cytokines and growth factors in 8293 healthy subjects of Finnish ancestry^25^, and the largest GWAS meta-analysis on stroke and stroke subtypes to date (MEGASTROKE)^26^.

Through checking the UKB SNP-Heritability Browser, we found the SNP-h2 for BC was 0.144. Hereby, to clarify whether genetically predicted circulating levels of cytokines are associated with risk of BC, we implemented two-sample MR analyses using data from this most comprehensive GWAS on cytokines^25^ and the largest BC GWAS from the Breast Cancer Association Consortium (BCAC) with totally 122,977 BC cases and 105,974 healthy controls^27^. Totally 24 cytokines with available data, including Eotaxin, growth-regulated oncogene-a (GROa), interleukin-12, p70 (IL12p70), interleukin-13(IL13), interleukin-16(IL16), monocyte chemoattractant protein-1/C-C motif chemokine ligand 2 (MCP1/CCL2), macrophage migration inhibitory factor (MIF), macrophage inflammatory protein-1 beta/C-C motif chemokine ligand 4 (MIP1b/CCL4), and stem cell factor (SCF), beta nerve growth factor (bNGF), cutaneous T-cell attracting (CTACK), hepatocyte growth factor (HGF), interleukin-17 (IL17), interleukin-18 (IL18), interleukin-2 receptor, alpha subunit (IL2ra), interferon gamma-induced protein 10 (IP10), macrophage colony-stimulating factor (MCSF), monokine induced by interferon-gamma (MIG), platelet derived growth factor BB (PDGFbb), regulated on activation, normal T cell expressed and secreted (RANTES), stem cell growth factor beta (SCGFb), tumor necrosis factor-beta (TNFb), TNF-related apoptosis inducing ligand (TRAIL), and vascular endothelial growth factor (VEGF), were evaluated for their associations with BC risk. We predicted the circulating cytokine levels using a set of estimated SNP effects based on a GWAS to identify protein quantitative trait loci (pQTL). Estrogen receptor (ER) status is important as a BC prognostic and predictive biomarker, and could also affect the medical decision of hormonal therapy or other treatments^28^. Thus, we also conducted stratified analyses of ER-positive and ER-negative BC.

## Results

### Overview outlines of the cytokines with BC risk

**Fig. 1:**
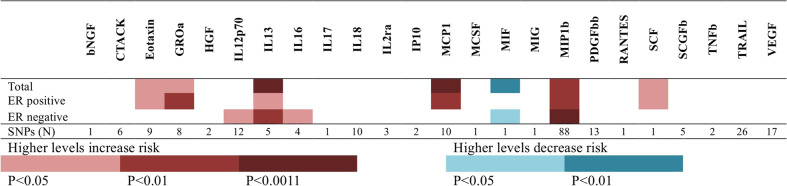
Mendelian randomization analyses of circulating cytokine and growth factor levels with risk of BC and subgroups.

In the current study, we evaluated whether genetically predicted circulating levels of cytokines are associated with risk of overall BC, ER-positive and ER-negative BC. Through filtering the threshold of significance (*p* < 5 × 10^−8^), false discovery rate (FDR < 5%), *F*-statistics (>10), and linkage disequilibrium (LD; *r*^2^ < 0.1), 229 independent SNPs were finally selected as the proxy of circulating levels of 24 cytokines. Figure 1 and Supplementary Table 1 presented the number of SNPs included in the genetic instruments, along with *R*^2^, *F*-statistics for the instruments, and the results of MR analyses of circulating levels of 24 cytokine with risk of BC and subgroups. *F*-statistics for their respective genetic instruments ranged from 29 to 636, suggesting that our analyses were unlikely to suffer from weak instrument bias. Among them, nine cytokines, including Eotaxin, GROa, IL12p70, IL13, IL16, MCP1, MIF, MIP1b, and SCF, showed significant associations with risk of BC in either total participants or subgroups (Fig. 1). Higher levels of eight cytokines was associated with increased risk of BC, while higher level of MIF was associated with decreased risk of BC. Even following Bonferroni correction for testing multiple cytokines (*p* < 0.05/48 = 1.1 × 10^−3^), three cytokines, including MCP1, MIP1b and IL13, still showed statistically significant associations with risk of total BC or subtype BC.

### Genetically predicted circulating levels of MCP1, MIP1b, and IL13 and risk of BC

**Fig. 2:**
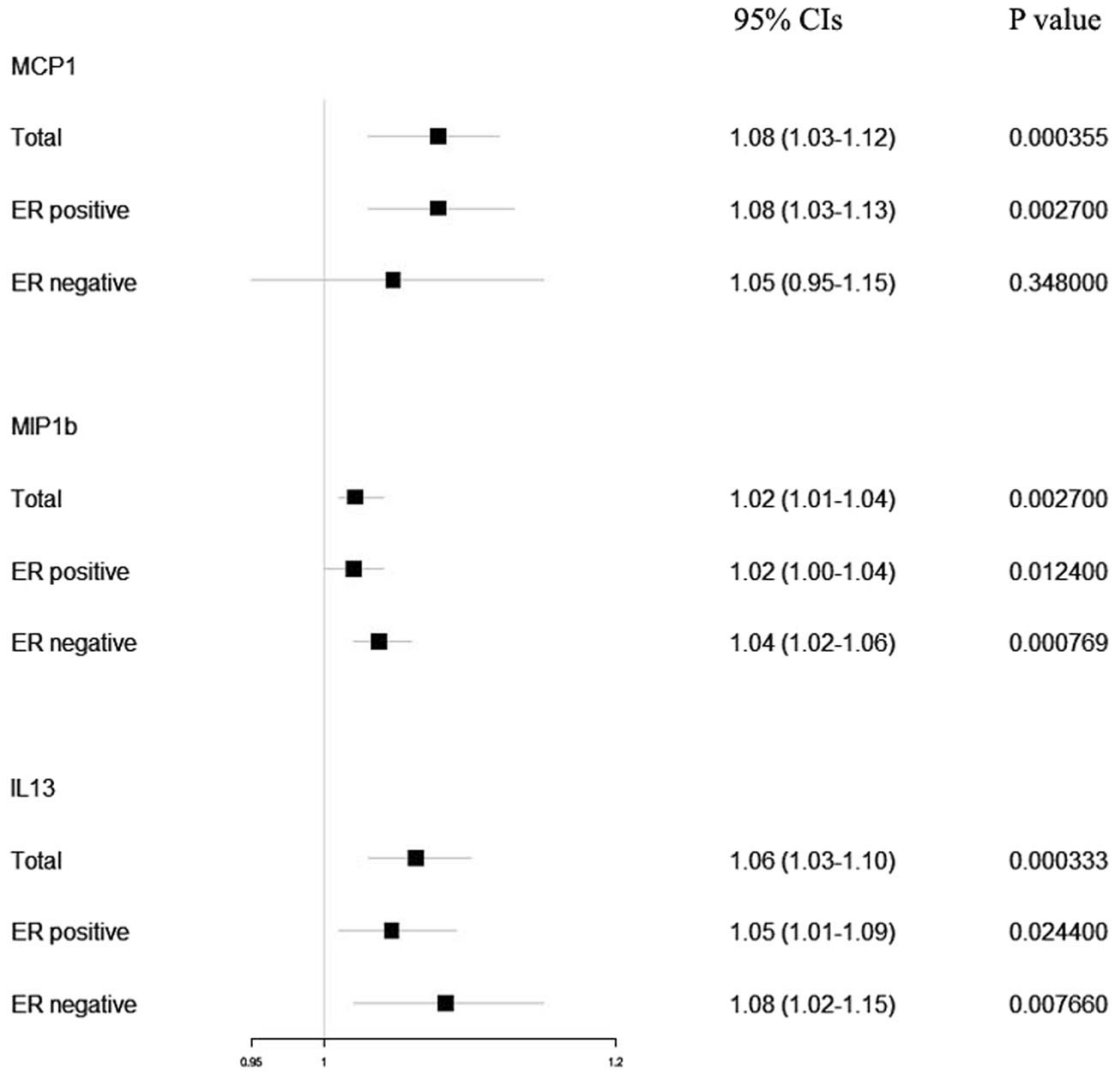
Causal estimates of selected cytokines (MCP1, MIP1b and IL13) on risk of overall, ER-positive and ER-negative breast cancer. Inverse-variance weighted (IVW) method was used as the primary method for the MR analyses to test the potential causal associations between Circulating Levels of selected cytokines and BC risk. Causal estimates express the change in odds ratio (OR) per standard deviation (SD) increment in cytokine concentration. Error bars indicate 95% confidence intervals.

**Fig. 3:**
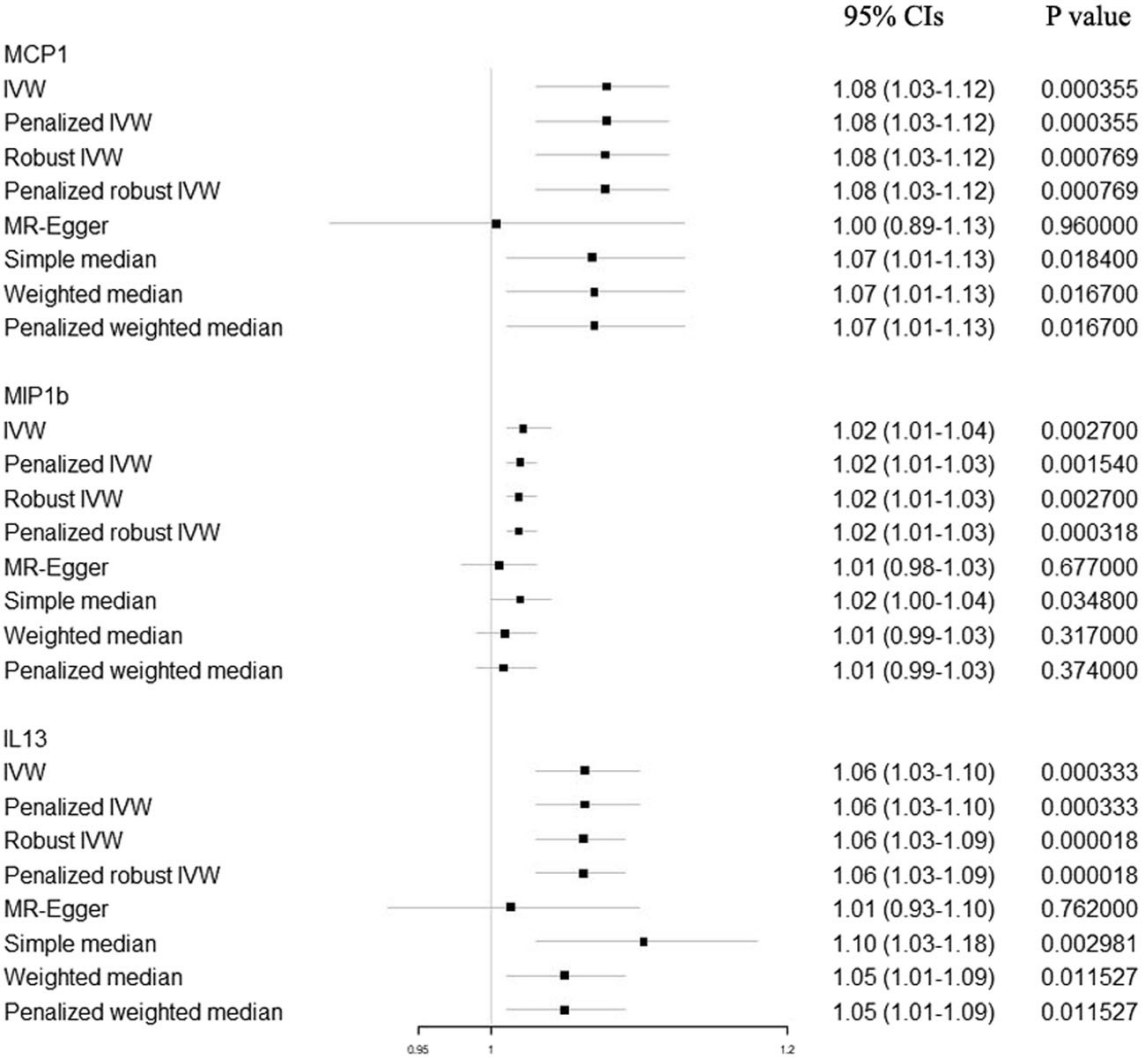
Alternative analyses (penalized IVW, robust IVW, penalized robust IVW, MR-Egger, simple median, weighted median, and penalized weighted median method) for causal estimates of selected cytokines (MCP1, MIP1b and IL13) on risk of breast cancer. Causal estimates express the change in odds ratio (OR) per standard deviation (SD) increment in cytokine concentration. Error bars indicate 95% confidence intervals.

The results of primary analyses for genetically predicted circulating levels (1-SD increase) of MCP1, MIP1b, and IL13 and risk of BC were presented in Fig. 2. In the primary analyses, genetically raised MCP1 (OR: 1.08; 95% CIs: 1.03–1.12; *P* value: 3.55 × 10^−4^), MIP1b (OR: 1.02; 95% CIs: 1.01–1.04; *P* value: 2.70 × 10^−3^) and IL13 (OR: 1.06; 95% CIs: 1.03–1.10; *P* value: 3.33 × 10^−4^) were associated with increased BC risk. For ER-positive BC cases, genetically raised MCP1 (OR: 1.08; 95% CIs: 1.03–1.13; *P* value: 2.70 × 10^−3^), MIP1b (OR: 1.02; 95% CIs: 1.00–1.04; *P* value: 0.012) and IL13 (OR: 1.05; 95% CIs: 1.01–1.09; *P* value: 0.024) were consistently associated with increased BC risk. For ER-negative BC cases, genetically raised MIP1b (OR: 1.04; 95% CIs: 1.02–1.06; *P* value: 7.69 × 10^−4^) and IL13 (OR: 1.08; 95% CIs: 1.02–1.15; *P* value: 7.69 × 10^−3^) were consistently associated with increased BC risk. In alternative analyses (Fig. 3), there was also strong evidence for associations of genetically raised levels of MCP1, MIP1b, and IL13 with BC risk, except for the MR-Egger method, which was mainly used to detect the possible pleiotropy effect. Cochran Q test for the heterogeneity in the MCP1 and IL13 associations with BC risk didn’t reject the null hypothesis (Cochran *P* value: 0.494 and 0.582, respectively). However, significant heterogeneity was detected for MIP1b (Cochran *P* value: 0.010). We also ran the MR analysis including the pleiotropic SNPs in the sensitivity analyses, and found results were not changed for IL13 and MIP1b. Genetically raised MCP1 (OR: 1.06; 95% CIs: 1.02–1.10; *P* value: 0.001) was still associated with increased BC risk, basing on 14 instrumental SNPs. During the bidirectional MR, results showed no evidence of BC causally influencing the levels of MCP1 (OR: 1.00; 95% CIs: 0.95–1.04; *P* value: 0.885), MIP1b (OR: 1.01; 95% CIs: 0.97–1.06; *P* value: 0.573) and IL13 (OR: 1.03; 95% CIs: 0.96–1.11; *P* value: 0.380).

### Detection of possible pleiotropy effect

To remove the potential influence of the possible pleiotropy effect, a series of measures have been adopted. At the stage of instrument construction, SNPs those were associated with levels of more than one cytokine were removed. At the stage of data analysis, first there was no indication for directional pleiotropy effects as assessed by the MR-Egger intercept for MCP1, MIP1b, and IL13 (*P* value: 0.200, 0.112, and 0.224, respectively). Second, MR-PRESSO analyses were conducted and revealed no potential outliers for MCP1 and IL13 (*P* value: 0.533 and 0.350, respectively). One outlier variant (rs7621046) was detected for MIP1b. Following exclusion of SNP rs7621046 in the restrictive MR analysis, genetically raised MIP1b was still associated with higher risk of any BC (OR: 1.02; 95% CIs: 1.01–1.03; *P* value: 1.80 × 10^−3^), while Cochran Q test for the heterogeneity didn’t reject the null hypothesis (Cochran *P* value: 0.151). Further, comprehensive lookup of the MR-Base, PhenoScanner and the GWAS catalog didn’t reveal significant associations of the selected variants in current study with other traditional BC risk factors, including height, obesity, age of menarche and menopause.

## Discussion

In this two-sample MR analysis with the largest GWAS datasets, we systematically screened 41 cytokines and identified that genetically predicted circulating levels (1-SD increase) of MCP1, MIP1b, and IL13 were all significantly associated with increased risk of overall BC and ER-positive BC. In addition, higher levels of MIP1b and IL13 were also significantly associated with increased risk of ER-negative BC. No obvious pleiotropy effect was detected. Besides, we also found suggestive evidence (which didn’t reaching the Bonferroni-corrected threshold) for associations between Eotaxin, GROa, IL12p70, IL16, MIF, SCF, and BC risk. To our knowledge, this study should be the largest and most comprehensive MR assessment of associations between genetically inflammatory cytokines and BC risk to date.

Genetically raised MCP1 was associated with increased overall BC risk (OR: 1.08; 95% CIs: 1.03–1.12; *P* value: 3.55 × 10^−4^), and ER-positive BC cases (OR: 1.08; 95% CIs: 1.03–1.13; *P* value: 2.70 × 10^−3^). Our systematic retrieval only identified limited observational studies reporting association between MCP1 level and BC risk. Some conclusions were null, possibly because our study had increased statistical power and previous reports might be false negative^29–33^. Some studies reported positive correlation between MCP1 level and BC risk, however, the conclusions were still underpowered or possibly biased due to the inherent defect of traditional observational studies^34–39^. Among the potentially modifiable cytokine factors, MIP1b which has the most instrumental SNPs, contributes to higher BC risk (OR: 1.02; 95% CIs: 1.01–1.04; *P* value: 2.70 × 10^−3^), especially in ER-negative BC cases (OR: 1.04; 95% CIs: 1.02–1.06; *P* value: 7.69 × 10^−4^). MIP1b plays a central role in the recruitment of regulatory T cells to B cells and antigen-presenting cells, and failure to do this would cause autoimmune activation^40^. IL13 is another cytokine associated with higher BC risk, especially in ER-negative BC cases (OR: 1.08; 95% CIs: 1.02–1.15; *P* value: 7.69 × 10^−3^). Autocrine IL13 plays an important role in the pathophysiology of BC, through inhibiting estrogen-induced proliferation and favoring acquisition of breast cancer cell differentiation markers^41^. Cytokines are vital to many biological processes, and its serum concentration is tightly regulated. We additionally found suggestive evidence for significant associations between Eotaxin, GROa, IL12p70, IL16, MIF, SCF, and BC risk. Among them, Eotaxin, GROa, IL12p70, IL16, and SCF took part in the regulation of inflammatory pathways in BC carcinogenesis, and contributed to higher BC risk^42–45^. Interestingly, genetically raised level of MIF was associated with decreased risk of BC (OR: 0.88; 95% CIs: 0.81–0.95; *P* value: 1.9 × 10^−3^), especially in ER-negative BC cases (OR: 0.84; 95% CIs: 0.72–0.97; *P* value: 0.018). This was contradictory to the results of observational studies, which showed higher MIF level in BC patients^46,47^. Possible reason might be the dual role of MIF in human BC carcinogenesis^48^. Further research is required to decipher the biological pathways underpinning these associations.

The methodological strength should be that we implemented the most recent and comprehensive dataset for cytokine levels and the largest available GWAS dataset for BC risk. The sample size in our analysis affords us greater power to detect causal associations with BC risk and allows us to more accurately estimate effect magnitudes. Indeed all the post-hoc powers for the primary associations of MCP1, MIP1b, and IL13 in overall BC, ER-positive BC and ER-negative BC have reached more than 90.0% at a significance level of 0.05. In addition, the post-hoc powers for the suggestive associations of Eotaxin (81.3%), GROa (76.0%), IL12p70 (71.2%%), IL16 (84.2%), MIF (91.6%) has also reached more than 70.0%. The interpretation and generalizability of the study findings are also affected by several limitations. First should be the limited cytokine instruments. To pursue the homogeneity of research cytokine variables, the instrument selection was based on a single GWAS. Although we implemented the most comprehensive and largest GWAS on cytokines, several cytokines being implicated in BC risk previously, such as CRP, TGF-β, were still missed in current analysis. Second, we didn’t obtain reliable genetic instruments for 17 cytokines as no SNPs meeting the strict filtering procedures. Third, we assumed the sex difference of cytokine was small in the current study, as no sex specific results were reported. Strictly speaking, the MR analyses in the study were testing the associations between female cytokines and female BCs. Fourth, we didn’t validate our findings in an independent GWAS, as there were no GWAS with sample sizes that match the BACA study (including 122,977 BC cases). Fifth, collider bias remains an issue in MR analysis. Sixth, we still can’t remove the possible pleiotropy effect (vertical or horizontal pleiotropy) that might be concealed by small sample size or the small number of SNP instruments, although we have used a series of methods, including MR-Egger, MR-PRESSO analyses, and checking whether the SNPs used as instruments have any known pleiotropic effects in curated genotype to phenotype databases. However, its impact is likely to be less than other biases, and it could be substantial only when the effects of the risk factor and confounders on selection are particularly large^49^. Sixth, the results from the MR-Egger analyses for MCP1, MIP1b, and IL13 were insignificant, which indicates the possibility of dependent on exclusion restriction.

Conclusively, using a two-sample MR approach, we find evidence that higher genetically predicted circulating level of MCP1, MIP1b, and IL13 are associated with increased risk of overall BC and ER-positive BC. These findings suggest the crucial role of cytokines in BC carcinogenesis and potential of targeting specific inflammatory cytokines for BC prevention. Further research is necessary to assess the viability of these cytokine biomarkers as drug targets for BC prevention and treatment. Nonetheless, these findings have potential implications for changing the inflammation status of the general women to reduce the burden of BC and improve female health.

## Methods

### Data source

For the genetic instruments of the cytokines, the summary statistics were taken from the most comprehensive and largest GWAS for cytokines, which genotyped up to 8293 Finnish participants from three independent population cohorts: the Cardiovascular Risk in Young Finns Study, FINRISK1997, and FINRISK2002^25^. For BC, we obtained the data from the largest BC consortia—BCAC, which provided summary association statistics for overall (105,974 controls and 122,977 BC cases), ER-positive (69,501 BC cases) and ER-negative BC (21,468 BC cases)^27^. The consortia results included the OncoArray (45,494 controls and 61,282 BC cases) and iCOGS datasets (42,892 controls and 46,785 BC cases), and the combined results from eleven additional GWAS (17,588 controls and 14,910 BC cases). To minimize the ancestral mismatch, current analyses are restricted to women of European ancestry only. All studies included rigorous quality control, imputation to the 1000 Genomes Project panel. As all analyses were based on summary statistics and not individual-level data, no ethical approval was required.

### Selection of cytokine instruments

The circulating cytokine levels were predicted using a set of estimated SNP effects based on a GWAS to identify pQTL. The full list of the 41 cytokines was provided in Supplementary Table 2^25^. For each cytokine, the single nucleotide polymorphisms (SNPs) were filtered according to the following procedures: (1) a genome-wide threshold of significance (*p* < 5 × 10^-8^) and a significance threshold of a FDR < 5% were adopted. This caused totally 7262 SNPs were selected. (2) To avoid the pleiotropic effect, 809 SNPs those were associated with levels of more than one cytokine were removed, leaving 6,453 SNPs for further step. (3) then, 5983 SNPs were identified to be available in the BCAC dataset. (4) We computed *F*-statistics to quantify the strength of the selected instruments, and all were well above the threshold of *F* statistics > 10 typically recommended for MR analyses. (5) Linkage disequilibrium (LD; *r*^2^ < 0.1 in the European 1000 G reference panel), which retaining SNPs with the lowest *p*-value as independent instrument, were exerted to remove the superposition effect of correlated SNPs using LDlink^50^. Finally, we identified 229 SNPs not in LD and to be significantly associated with circulating cytokine levels. These instruments related to the circulating levels of 24 cytokines, whereas for 17 cytokines no SNPs meeting the above conditions (Supplementary table 1).

### Statistical analyses

In current study, *R*^2^ representing the proportion of variance in a risk factor explained by the genetic instrument, and *F*-statistic representing the strength of the association between the genetic instrument and levels of the risk factor were calculated^51^. For cytokine exposures, we scaled MR estimates per standard deviation (SD) difference of the risk factor, as all effect sizes were in SD-scaled units in Ahola-Olli’s report^25^. Causal estimates are thus presented per genetically predicted SD, and a log-linear association with odds of BC is implicit across the range of intermediate risk exposure. In current study, inverse-variance weighted (IVW) method was used as the primary method for the MR analyses to test the potential causal associations between circulating levels of selected cytokines and BC risk. In the absence of directional pleiotropy, it provides robust causal estimates. Further, penalized IVW, robust IVW, penalized robust IVW, MR-Egger, simple median, weighted median, and penalized weighted median method were used for alternative analyses. Among these methods, MR-Egger allows free estimation of the intercept, and a statistically significant intercept term implies the presence of unbalanced pleiotropy and causal estimates in MR Egger are less precise than those in IVW. Meanwhile, we also conducted bidirectional MR to test the potential causal effect of BC (Supplementary Table 3 presents SNPs that were used as instruments for breast cancer) on cytokine levels. To detect the possible pleiotropy effect, we first tested whether the intercept from MR-Egger regression differed from zero, which provided evidence of directional pleiotropy. Second, the MR-Pleiotropy Residual Sum and Outlier (MR-PRESSO) was used to identify and correct for potential outliers^52^. Third, we looked up the MR-Base, PhenoScanner database and the GWAS catalog for potential associations of the selected variants in our study with other BC risk factors. Post-hoc power calculations were conducted for our IVW analyses using an online Mendelian randomization power calculation tool (https://sb452.shinyapps.io/power/)^53^. All statistical analyses were conducted using the R (version 3.6.3) or SAS 9.4 statistical software. All *P* values are two-tailed, and *P* < 0.05 was considered significant.

### Reporting summary

Further information on research design is available in the Nature Research Reporting Summary linked to this article.

## Supplementary information

SUPPLEMENTAL MATERIALs

Reporting Summary Checklist FLAT

## Data Availability

All summary genetic association data used in this study are available online, Cytokine GWAS and BCAC.
