# Supplementary material for: Mendelian randomization analyses of genetically predicted circulating levels of cytokines with risk of breast cancer
Source: NPJ Precis Oncol. 2020 Sep 1;4:25. doi: 10.1038/s41698-020-00131-6 (PMC7462857; doi:10.1038/s41698-020-00131-6)
Supplement: Supplementary file 1 — SUPPLEMENTAL MATERIALs [file 41698_2020_131_MOESM1_ESM.pdf]

Supplementary table 1 Characteristics of the genetic instruments for the cytokines in the Mendelian randomization study

Cytokines	SNPs (N)	Variance explained (R^2)	F-statistic [range]	Total samples		Oncoarray		iCOGS		GWAS		ER Positive		ER Negative	
				Beta	Se	Beta	Se	Beta	Se	Beta	Se	Beta	Se	Beta	Se
bNGF	1	0.010	34	0.014	0.044	-0.014	0.064	0.019	0.069	0.103	0.125	0.024	0.052	-0.089	0.081
CTACK	6	0.088	30-75	-0.004	0.015	-0.007	0.022	0.007	0.026	-0.025	0.043	0	0.018	-0.011	0.028
Eotaxin	9	0.042	31-61	0.054	0.023	0.062	0.032	0.03	0.05	0.097	0.068	0.054	0.027	0.07	0.047
GROa	8	0.143	30-115	0.029	0.013	0.019	0.025	0.053	0.02	-0.006	0.035	0.038	0.014	0.04	0.028
HGF	2	0.013	32-34	0.022	0.04	0.038	0.056	0.023	0.064	-0.048	0.117	0.01	0.047	0.116	0.073
IL12p70	12	0.060	32-55	0.033	0.028	0.039	0.039	0.027	0.04	0.03	0.054	0.013	0.032	0.079	0.038
IL13	5	0.100	32-211	0.061	0.017	0.063	0.033	0.057	0.031	0.061	0.041	0.045	0.02	0.08	0.03
IL16	4	0.083	30-60	0.019	0.017	0.026	0.033	-0.013	0.029	0.068	0.046	0.015	0.02	0.074	0.031
IL17	1	0.004	31	0.048	0.073	0.157	0.098	-0.106	0.132	-0.041	0.191	0.043	0.087	0.115	0.129
IL18	10	0.101	31-45	0.02	0.019	-0.008	0.024	0.05	0.025	0.043	0.045	0.03	0.022	0.037	0.028
IL2ra	3	0.039	32-45	0.051	0.023	0.076	0.038	0.025	0.035	0.047	0.064	0.069	0.027	0.024	0.041
IP10	2	0.020	31-32	0.028	0.034	0	0.047	0.095	0.057	-0.059	0.104	0.005	0.041	0.041	0.062
MCP1	10	0.047	30-66	0.075	0.021	0.104	0.04	0.018	0.033	0.143	0.062	0.075	0.025	0.046	0.049
MCSF	1	0.017	32	-0.027	0.036	-0.042	0.05	0.017	0.061	-0.081	0.099	-0.014	0.043	-0.114	0.065
MIF	1	0.012	39	-0.127	0.041	-0.171	0.059	-0.065	0.065	-0.152	0.116	-0.089	0.049	-0.178	0.075
MIG	1	0.008	34	0.058	0.05	0.008	0.072	0.134	0.08	0.005	0.149	0.117	0.06	-0.058	0.094
MIP1b	88	0.756	30-636	0.021	0.007	0.025	0.009	0.012	0.009	0.033	0.016	0.02	0.008	0.037	0.011
PDGFbb	13	0.069	30-77	0.017	0.018	0.021	0.026	0.008	0.041	0.031	0.053	0.022	0.024	0.027	0.043
RANTES	1	0.007	30	-0.079	0.054	-0.094	0.08	0.02	0.084	-0.35	0.152	-0.125	0.065	-0.051	0.099
SCF	1	0.004	31	0.135	0.066	0.062	0.097	0.256	0.106	0.024	0.188	0.19	0.079	0.036	0.121
SCGFb	5	0.063	34-63	0.032	0.035	0.036	0.035	-0.006	0.035	0.186	0.062	0.011	0.036	0.066	0.034
TNFb	2	0.095	35-80	0.019	0.017	0.032	0.022	0.007	0.029	-0.017	0.051	0.024	0.02	-0.012	0.03

TRAIL	26	0.292	30-352	-0.007	0.012		-0.007	0.014		0.006	0.016		-0.043	0.033		-0.015	0.013		-0.007	0.019
VEGF	17	0.092	29-53	0.018	0.017		0.012	0.032		0.027	0.027		0.018	0.045		0.004	0.02		0.061	0.03

R^2 represents the proportion of variance in a risk factor explained by the genetic instrument. F-statistic represents the strength of the association between the genetic instrument and levels of the risk factor.

Supplementary table 2 Cytokines examined by the original GWAS study and number of genetic instruments (SNPs) identified from our approach.

Cytokines	Total N	SNPs (N)
bNGF	3531	1
CTACK	3631	6
Eotaxin	8153	9
FGFBasic	7565	0
GCSF	7904	0
GROa	3505	8
HGF	8292	2
IFNg	7701	0
IL-10	7681	0
IL-12p70	8270	12
IL-13	3557	5
IL-16	3483	4
IL-17	7760	1
IL-18	3636	10
IL-1b	3309	0
IL1ra	3638	0
IL-2	3475	0
IL2ra	3677	3
IL-4	8124	0
IL-5	3364	0
IL-6	8189	0
IL-7	3409	0
IL-8	3526	0
IL-9	3634	0
IP10	3685	0
MCP1	8293	10
MCP3	843	0
MCSF	840	1
MIF	3494	1
MIG	3685	1
MIP1a	3522	0
MIP1b	8243	88
PDGFbb	8293	13
RANTES	3421	1
SCF	8290	1
SCGFb	3682	5
SDF1a	5998	0
TNFa	3454	0
TNFb	1559	2
TRAIL	8186	26

VEGF	7118	17

Supplementary table 3 SNPs that were used as instruments for breast cancer

SNP	effect_allele	other_allele	beta	se	pval
rs10022462	T	C	0.0375	0.0062	1.55E-09
rs10054203	C	G	0.0383	0.0065	3.21E-09
rs10096351	G	A	0.1055	0.0062	1.97E-64
rs10623258	CTT	C	0.0354	0.0063	2.26E-08
rs10760444	A	G	-0.0358	0.0062	9.06E-09
rs10885405	T	C	0.0465	0.0062	5.44E-14
rs10941679	G	A	0.1278	0.0071	5.61E-73
rs10978911	C	G	0.0839	0.0091	2.36E-20
rs10995201	G	A	-0.1317	0.0087	1.56E-51
rs11117758	A	G	-0.0449	0.0076	3.90E-09
rs11135046	T	G	-0.0717	0.0062	4.68E-31
rs11205303	C	T	0.0497	0.0064	1.14E-14
rs1121948	G	A	0.0575	0.0077	7.48E-14
rs11249433	G	A	0.0988	0.0065	1.76E-52
rs11462858	GT	G	-0.0471	0.0067	2.09E-12
rs11571833	T	A	0.2727	0.0346	3.10E-15
rs11583393	A	C	-0.0396	0.0072	3.61E-08
rs11624333	C	T	-0.0968	0.0071	6.50E-42
rs11672660	T	C	0.0472	0.008	4.13E-09
rs11684853	T	G	-0.0442	0.0062	1.12E-12
rs117618124	C	T	-0.1074	0.0156	5.46E-12
rs117737783	A	G	0.0747	0.0127	4.03E-09
rs11822830	G	A	0.0483	0.0063	1.98E-14
rs11903787	A	G	-0.0451	0.0076	2.36E-09
rs11977670	A	G	0.0522	0.0063	1.05E-16
rs12207986	A	G	0.0375	0.0062	1.45E-09
rs12250948	C	T	-0.0576	0.0074	1.04E-14
rs1230666	G	A	-0.0592	0.0095	3.87E-10
rs12479355	G	A	-0.0426	0.0076	2.36E-08
rs12487185	G	A	0.045	0.0066	1.21E-11
rs12513872	A	G	0.0413	0.0073	1.46E-08
rs12519859	A	G	0.0365	0.0063	5.77E-09
rs12546444	T	A	-0.0717	0.011	7.45E-11
rs1268974	G	A	-0.0793	0.0063	3.71E-36
rs12990503	G	C	-0.073	0.007	9.99E-26
rs13066793	G	A	-0.0685	0.0112	1.04E-09
rs13267382	G	A	-0.0437	0.0065	1.60E-11
rs1498608	A	T	-0.0671	0.0107	3.48E-10
rs150658557	TA	T	-0.0605	0.0083	2.30E-13
rs1685191	T	C	-0.0586	0.0064	3.87E-20
rs16991615	A	G	0.0758	0.0126	1.92E-09
rs1707302	G	A	0.0364	0.0066	2.95E-08

rs170801	A	C	-0.0591	0.007	3.18E-17
rs17156577	C	T	0.0578	0.0098	4.25E-09
rs17185310	A	G	0.0395	0.0069	8.70E-09
rs17268829	C	T	0.0495	0.0068	4.49E-13
rs17426269	A	G	0.0487	0.0086	1.74E-08
rs1778523	G	C	-0.0384	0.0066	7.55E-09
rs1973765	C	T	-0.0817	0.0064	9.71E-38
rs1985742	T	A	0.0588	0.0065	8.10E-20
rs2016394	A	G	-0.0425	0.0062	6.23E-12
rs2253012	T	C	0.0433	0.0065	3.13E-11
rs2403907	A	C	-0.0796	0.0067	1.87E-32
rs2432539	G	A	-0.0349	0.0064	4.02E-08
rs2506889	T	C	-0.0615	0.0067	2.38E-20
rs2747652	C	T	0.0663	0.0062	1.31E-26
rs2787486	C	A	-0.0758	0.0068	5.56E-29
rs28512361	A	G	0.0611	0.0109	2.25E-08
rs2853669	G	A	-0.0651	0.0069	4.05E-21
rs2912779	C	T	-0.2374	0.0063	1.79E-313
rs2965183	A	G	0.0445	0.0065	6.31E-12
rs2992756	C	T	-0.0506	0.0063	1.60E-15
rs332529	A	G	-0.0606	0.0089	9.02E-12
rs34195518	CT	C	0.0611	0.0096	2.01E-10
rs35383942	T	C	0.101	0.0139	3.79E-13
rs35910339	G	C	0.0462	0.0067	5.41E-12
rs35961416	CA	C	-0.0417	0.0066	2.71E-10
rs3769821	T	C	-0.0565	0.0065	3.97E-18
rs3821902	G	T	0.0623	0.0089	2.99E-12
rs3903072	T	G	-0.0434	0.0062	2.25E-12
rs4081859	A	G	0.0521	0.0073	7.08E-13
rs418053	C	G	-0.0465	0.0063	1.20E-13
rs4233486	T	C	0.0396	0.0069	9.09E-09
rs4286946	G	C	-0.0799	0.0083	8.63E-22
rs4442975	T	G	-0.1274	0.0061	1.14E-95
rs4496150	A	C	-0.0416	0.0072	8.09E-09
rs4562056	T	G	0.0416	0.0067	4.72E-10
rs4702131	C	T	-0.0424	0.0062	1.00E-11
rs4784227	T	C	0.2153	0.0071	6.78E-201
rs4820318	A	G	-0.0475	0.0064	1.03E-13
rs4848599	C	T	0.0933	0.01	1.59E-20
rs4971059	A	G	0.0424	0.0064	4.83E-11
rs527616	G	C	0.0499	0.0064	6.70E-15
rs532436	A	G	0.0431	0.0078	2.91E-08
rs56013747	C	A	-0.0497	0.009	3.74E-08
rs56387622	C	T	-0.0942	0.0106	5.47E-19

rs56891541	TCC	T	0.0535	0.0072	8.99E-14
rs56971155	T	C	-0.0424	0.0064	3.63E-11
rs57212245	A	G	-0.0652	0.0119	4.09E-08
rs58058861	A	G	0.0474	0.0074	1.91E-10
rs5995875	T	C	0.1206	0.0098	9.04E-35
rs5997389	A	G	0.0791	0.0108	2.19E-13
rs60954078	G	A	0.1817	0.0117	2.84E-54
rs6122906	G	A	0.0507	0.008	2.45E-10
rs62048402	A	G	-0.0624	0.0063	3.50E-23
rs62237573	T	C	0.4256	0.034	6.87E-36
rs62331150	T	G	0.0494	0.0074	1.85E-11
rs62355901	C	T	0.1734	0.0082	3.42E-98
rs62485509	T	G	-0.0545	0.0079	4.45E-12
rs630965	T	C	0.0992	0.0064	3.21E-54
rs6472903	T	G	0.0778	0.0083	4.43E-21
rs6562760	G	A	0.0443	0.0073	1.49E-09
rs6569648	T	C	0.0512	0.0073	2.98E-12
rs6596100	T	C	-0.0439	0.0076	7.74E-09
rs6597981	G	A	0.0439	0.0062	1.35E-12
rs66572733	G	C	0.095	0.0162	4.74E-09
rs67087079	A	G	0.0527	0.0093	1.32E-08
rs6725517	G	A	-0.0468	0.0067	2.93E-12
rs6787391	T	C	0.0567	0.0064	9.07E-19
rs68056147	A	G	0.0501	0.0069	5.36E-13
rs6815814	C	A	0.052	0.0072	6.13E-13
rs6882649	T	G	0.0388	0.0066	3.67E-09
rs7072776	G	A	-0.0618	0.0068	1.75E-19
rs7149262	A	C	-0.0724	0.0076	2.47E-21
rs71557345	A	G	-0.0806	0.0129	3.87E-10
rs71559437	A	G	-0.0686	0.0099	5.05E-12
rs7223535	A	G	-0.0439	0.007	3.53E-10
rs7258465	C	T	-0.0725	0.0066	2.79E-28
rs72658071	T	A	0.1182	0.0107	2.58E-28
rs72725173	A	G	0.0559	0.0085	3.88E-11
rs72755295	G	A	0.1376	0.0179	1.65E-14
rs73949122	G	C	-0.0593	0.0084	1.85E-12
rs7500067	G	A	0.0785	0.0073	4.06E-27
rs7626742	T	G	0.1046	0.0062	1.61E-63
rs7650602	C	T	0.0489	0.0063	6.22E-15
rs76956704	T	C	0.1352	0.0182	1.27E-13
rs7697216	C	T	0.1039	0.0098	1.61E-26
rs77528541	T	G	-0.0583	0.0096	1.41E-09
rs77554484	A	C	-0.0741	0.0093	1.28E-15
rs78381082	G	A	-0.0611	0.0089	5.43E-12

rs78425380	C	T	0.0603	0.0099	1.03E-09
rs78440108	T	C	-0.0595	0.0084	1.62E-12
rs78540526	T	C	0.2758	0.0113	1.81E-131
rs7904249	A	G	0.0764	0.0084	6.98E-20
rs7971	G	A	-0.0365	0.0065	1.93E-08
rs8082452	G	T	-0.0418	0.0065	1.14E-10
rs9284657	G	C	0.0366	0.0062	3.36E-09
rs9361840	G	A	0.0541	0.0072	7.14E-14
rs9693444	C	A	-0.0626	0.0066	1.60E-21
rs9833888	T	G	0.0457	0.0074	5.15E-10
rs9952980	C	T	-0.0478	0.0065	1.65E-13
rs9954058	C	G	-0.0891	0.0122	2.79E-13
